# Non-edible onion skin waste as a source of bioactive agents for functional foods development: Chemical composition and multifunctional bioactivity

**DOI:** 10.1016/j.fochx.2025.102794

**Published:** 2025-07-17

**Authors:** Esther Trigueros, Óscar Benito-Román, Andreia P. Oliveira, Romeu A. Videira, Eugénia Pinto, Paula B. Andrade, M. Teresa Sanz, Sagrario Beltrán

**Affiliations:** a REQUIMTE/LAQV*,* Laboratório de Farmacognosia, Departamento de Química, Faculdade de Farmácia, Universidade do Porto, R. Jorge Viterbo Ferreira, n° 228, 4050-313 Porto, Portugal; b Department of Biotechnology and Food Science, Chemical Engineering Division, University of Burgos, Plza. Misael Bañuelos s/n 09001, Burgos, Spain; c Associate Laboratory i4HB – Institute for Health and Bioeconomy, University Institute of Health Sciences – IUCS-CESPU, 4585-116 Gandra, Portugal; d UCIBIO – Research Unit on Applied Molecular Biosciences, Translational Toxicology Research Laboratory, University Institute of Health Sciences (1H-TOXRUN, IUCS-CESPU), 4585-116 Gandra, Portugal; e Laboratory of Microbiology, Biological Sciences Department, Faculty of Pharmacy, University of Porto, 4050-313 Porto, Portugal; f Interdisciplinary Centre of Marine and Environmental Research (CIIMAR), University of Porto, 4450-208 Matosinhos, Portugal

**Keywords:** Onion waste, Quercetin, Cell assays, Antioxidant, Antidiabetic, Anti-inflammatory, Functional foods

## Abstract

Onion skin, often discarded during processing, remains rich in bioactive compounds, particularly flavonoids. This study assessed phenolic extraction (37 °C, 60 min) from *Horcal* and *Red* cultivars, using 70:30 ethanol:water. Extracts were rich in phenolics (103–155 mg/g), mainly quercetin and quercetin-4’-*O*-glucoside (61–67 %), and showed strong O_2_^●-^ (IC_50_ = 26–27.7 μg/mL) and ^●^NO (IC_50_ = 53–56 μg/mL) scavenging. They also inhibited α-glucosidase (IC_50_ = 2.2–2.3 μg/mL) and aldose reductase (IC_50_ = 37–44 μg/mL), while preserving α-amylase activity (IC_50_ = 932–1126 μg/mL). Both extracts demonstrated anti-inflammatory effects via 5-LOX inhibition (IC_50_ = 30.2–47 μg/mL) and anti-neuroinflammatory effects by restoring NO in BV2 cells. They further inhibited tyrosinase (IC_50_ = 14.9–15.5 μg/mL) and xanthine oxidase (IC_50_ = 15.1–19.2 μg/mL) above acetylcholinesterase (IC_50_ = 301–532 μg/mL), with *Horcal* showing greater potential due to higher phenolic content. Extracts were non-cytotoxic and displayed anti-dermatophytic activity, particularly against *T. rubrum* and *E. floccosum*. These results suggest onion skin as a promising resource for bioactive compounds, with potential applications in supplements, functional foods, and nutraceuticals.

## Highlights

• Onion skin extracts were rich in quercetin and its glucosides (69–107 mg/g).
• Extracts strongly scavenged O_2_^●-^ and ^●^NO (IC_50_: 26–27.7 and 53–56 μg/mL, resp).
• Antidiabetic effects were confirmed by inhibiting α-glucosidase and aldose reductase.
• Anti-inflammatory effects were shown by 5-LOX inhibition and NO level restoration.
• Extracts were non-cytotoxic and showed anti-dermatophytic effects.

## 1 Introduction

Onion (*Allium cepa* L.) is one of the most significant vegetable crops worldwide, with production increasing due to rising consumption, driven by its nutritional, medicinal, and functional properties. Onion processing generates around 37 % waste, mainly in the form of peels and skins, which are usually discarded in landfills, raising environmental concerns (Sharma et al., 2016). Nevertheless, onion skin, a by-product of industrial peeling, is rich in bioactive compounds, especially phenolics and flavonoids, with quercetin and quercetin-4´-*O*-glucoside as major compounds, accompanied by other quercetin degradation and oxidation products (Kumar et al., 2022). Despite their bioactive potential, these compounds are largely underutilized after onion processing, thus missing a valuable resource. These compounds are found in higher concentrations in onion skin than in the edible bulb (Sharma et al., 2016) and are associated with a range of bioactivities, including cancer prevention, obesity management, antidiabetic effects, neuroprotection, cardiovascular health, and antimicrobial properties (Kumar et al., 2022). In this sense, valorizing onion skin could advance Sustainable Development Goals (SDGs) 2 and 12 of the 2023 Agenda (United Nations, 2015), promoting sustainable agriculture and production, and providing access to new natural sources of nutritional and bioactive compounds.

To date, most research has focused on the chemical and biological properties of the edible onion bulb, while the bioactive potential of the non-edible onion skin has been largely overlooked. Only a few studies have investigated the antioxidant properties of onion skin, typically focusing on non-biological radicals, such as DPPH radical scavenging and Fe^3+^/Fe^2+^ reducing power assays (Khalili et al., 2022). Evaluation of their bioactive potential using cellular models remains limited, and the link between main compounds in onion skin composition and the observed biological effects has not been previously evaluated. Therefore, this study aims to fill the exiting gap in the literature by extracting flavonoid-rich compounds from onion skin waste (*Horcal* and *Red* cultivars) and evaluating their bioactive properties to clarify the link between their composition and biological effects. The specific objectives include: (1) to assess the chemical composition of the onion skins, (2) to examine their antioxidant activity against biological oxidants, (3) to evaluate their antidiabetic and anti-inflammatory properties in cellular and non-cellular models, (4) to investigate their enzyme inhibitory effects, (5) to test the extract cytotoxicity and antimicrobial activity, and (6) to compare with the bioactivity of major compounds in the extracts to establish a composition-bioactivity relationship.

## 2 Materials and methods

### 2.1 Chemical reagents

2-mercaptoethanol, 3,5-dinitrosalicylic acid, 4-nitrophenyl-*α*-D-glucopyranoside, γ-IFN (≥ 98 %), acarbose (95 %), acetylthiocholine iodide (ATCI), allopurinol (≥ 99 %), bovine serum albumin (BSA) (96 %), D,L-glyceraldehyde, gallic acid (≥ 98.0 %), galanthamine hydrobromide from *Lycoris* sp*.* (≥ 94 %), isorhamnetin (≥ 95.0 %), kaempferol (≥ 97.0 %), kojic acid (≥ 98.5 %), L-DOPA, linoleic acid, myricetin (≥ 96.0 %), *N*-(1-naphthyl)ethylenediamine dihydrochloride, NADH, nitrotetrazolium blue chloride, *p*-coumaric acid (≥ 98 %), phenazine methosulfate, protocatechuic acid (≥ 98.0 %), quercetin (95 %), quercetin-3,4´-*O*-diglucoside (≥ 85 %), quercetin-3-*O*-glucoside (≥ 98 %), quercetin-4´-*O*-glucoside (≥ 95.0 %), sodium nitroprusside dihydrate, starch, sucrose (≥ 99.0 %), sulfanilamide, trypan blue solution (0.4 %) and xanthine (≥ 99 %) supplied by Sigma-Aldrich (St. Louis, MO, USA); fetal bovine serum (FBS), Dulbecco's Modified Eagle Medium DMEM (1×) + GlutaMAX, AB “Pen Strep” Glico, Hank's balanced salt solution (HBSS) and trypsin by Gibco™, Thermo Fisher (Waltham, MA, USA); NADPH by Prozomix (UK); 5–5’-Dithiobis(2-nitrobenzoic acid) (DTNB) and MTT by Alfa Aesar Chemicals (Haverhill, MA, USA). Enzymes for *in vitro* enzymatic assays were aldose-reductase (*Homo sapiens,* human) from Prozomix (UK), α-amylase (porcine pancreas), α-glucosidase (*Saccharomyces cerevisiae*), acetylcholinesterase from *Electrophorus electricus*, lipoxygenase from *Glycine* max (soybean), tyrosinase from mushroom, xanthine oxidase from bovine milk, all provided by Sigma-Aldrich (St. Louis, MO, USA).

### 2.2 Samples

Onion (*Allium cepa* L.) skin samples correspond to two different onion cultivars: namely *Horcal* and *Red*, supplied by “Embutidos Cardeña” (Burgos, Spain). The outermost skins were separated, air-dried at room temperature (25 °C), and milled (SM100 Retsch mill, 1 mm sieve).

### 2.3 Extraction procedure

Extraction was conducted at 37 °C for 60 min using an ethanol:water (70:30, v/v) mixture as solvent, based on the optimal conditions identified in a previous study (Benito-Román et al., 2021). Briefly, 50 g of onion skin were transferred to an Erlenmeyer flask, and 400 mL of solvent were added. The flask was placed in an incubator shaker (Model G25, New Brunswick Scientific Co., NJ, USA) and stirred at 275 rpm. After the extraction, the solid liquid mixture was separated under vacuum filtration. Solids were discarded and the ethanol was removed from the liquid extract in a rotary evaporator (Heidolph Laborota 4001) at 37 °C and pressure below 100 mbar. The ethanol-free extracts were frozen at −80 °C and freeze-dried (Labconco Freeze Dry System) at 0.15 mbar for at least 48 h to get a dry powder, which was stored for later use. For chemical composition analysis, *in vitro* antioxidant activity assays, and non-cellular enzymatic assays, the freeze-dried extracts were reconstituted in the original extraction solvent (70 % ethanol). For cellular and microbiological assays, DMSO was used as the reconstitution solvent, as detailed further below.

### 2.4 Chemical composition

The freeze-dried powders were reconstituted in 70 % ethanol to obtain the “stock solution” (110 mg/mL), which was diluted as needed to fit within the calibration curves used for each determination and was referred to as the “extract solution”. The phenol‑sulfuric method (Nielsen, 2010) with slight variations was employed to determine sugars in the hydroalcoholic extracts. Equal volumes (100 μL) of the extract solution and phenol solution were mixed with five volumes of sulfuric acid (6.5 %, v/v). Absorbance was measured at 490 nm. Sucrose was used for the calibration curve. The protein content was measured by mixing the extract solution (40 μL) with Bradford reagent (200 μL). After 5 min in the dark, absorbance was measured at 595 nm (Bradford, 1976). A standard curve was created using BSA. Total flavonoid content (TFC) was determined following the method described by Chang et al. (2002). The extract solution was mixed with 0.1 M potassium acetate, (10 %, w/*v*) aluminum chloride, and distilled water, followed by a 30 min incubation. After filtering (0.45 μm), absorbance was recorded at 415 nm. Quercetin was used for constructing the calibration curve. Total phenolic content (TPC) was determined by mixing 1 mL of extract solution with 1 mL Folin-Ciocalteau reagent, 2 mL of 25 % (w/*v*) sodium carbonate, and 3 mL of distilled water. The mixture was incubated in the dark for 60 min, and the absorbance was measured at 750 nm (Trigueros, Oliveira, et al., 2024). Gallic acid was used as the standard for calibration. Phenolic compounds identification and quantification was carried out using High-Performance Liquid Chromatography (Agilent 1100, CA, USA) equipped with a Diode Array Detector (HPLC-DAD), and a Kinetex® Biphenyl column (Phenomenex). The acquisition parameters were previously described (Trigueros, Benito-Román, et al., 2024). Analyses were performed in triplicate.

### 2.5 *In vitro* radical scavenging activity

The freeze-dried onion skin extracts were reconstituted in 70 % ethanol (“stock solution” - 110 mg/mL) and serially diluted to obtain the “extract solution” at various concentrations, allowing the assessment of a full range of scavenging activity, from maximum (100 %) to minimal level, to determine the IC_50_ values.

#### 2.5.1 Nitric oxide scavenging activity

The capacity of the onion skin extracts to scavenge the nitric oxide (^●^NO) radical was assessed using the method described by Ferreres et al. (2021). This involved mixing 100 μL of the extract solutions, diluted in 0.1 M phosphate buffer (pH 7.4), with the same volume of 20 mM sodium nitroprusside dihydrate. After a 60 min incubation under light, the Griess reagent (100 μL) was added, followed by 10 min incubation in the dark, and absorbance was measured at 560 nm. Quercetin was used as positive control. At least five different concentrations were tested for each onion skin extract, with a minimum of three independent experiments conducted in triplicate. A control was prepared replacing sample with buffer, and a blank with phosphoric acid instead of the Griess reagent.

#### 2.5.2 Superoxide scavenging activity

The ability of the onion skin extracts to scavenge the superoxide anion (O_2_^●-^) radical was determined according to Ferreres et al. (2021). Equal volumes (50 μL) of the extract solutions, diluted in 19 mM phosphate buffer (pH 7.4), and 0.166 mM NADH, and three volumes of 0.043 mM nitrotetrazolium blue chloride were combined. The reaction was initiated by adding 50 μL of 2.7 μM phenazine methosulfate, and absorbance was recorded at 560 nm. A control was prepared substituting sample with buffer.

### 2.6 Cell-free enzymatic assays

As previously described, the freeze-dried onion skin extracts were reconstituted in 70 % ethanol to prepare a 110 mg/mL stock solution, which was serially diluted to obtain the “extract solutions” for assessing enzyme inhibition across a full activity range and determining the IC_50_ values.

#### 2.6.1 α-Amylase activity assay

To evaluate the inhibitory effect of the extracts and pure compounds on pancreatic α-amylase activity, 200 μL of extract solutions in 20 mM phosphate buffer (pH 6.9) and 100 μL of 1 % (w/*v*) starch were incubated for 10 min at 25 °C. This was followed by the addition of 100 μL of pancreatic amylase solution and incubation. Next, 200 μL of dinitrosalicylic acid were added, and the mixture was incubated at 100 °C for 5 min. After cooling, the mixture was diluted with distilled water (1:2.5, v/v), and absorbance was measured at 540 nm. A control was prepared using buffer instead of sample, and a blank was prepared for each extract solution concentration by replacing the enzyme solution with buffer (Ferreres et al., 2021).

#### 2.6.2 Aldose reductase activity assay

Aldose reductase activity was assessed following the method developed by Ferreres et al. (2021) with minor adjustments. Equal volumes (40 μL) of the extract solutions, aldose reductase, and 10 mM D,L-glyceraldehyde were incubated at 37 °C for 2 min. Reaction was initiated by adding 80 μL of 500 mM NADPH, with absorbance measured at 340 nm immediately, and after a 20 min incubation. A control was prepared by substituting the sample with 100 mM phosphate buffer (pH 6.2).

#### 2.6.3 Α-Glucosidase activity assay

α-Glucosidase activity was evaluated incubating 180 μL of a mixture of the extract solutions, prepared in 10 mM potassium phosphate buffer (pH 7.0), and 20 μL of the enzyme solution at 37 °C for 2 min. Absorbance at 405 nm was measured after adding 100 μL of 2.5 mM 4-nitrophenyl-α-D-glucopyranoside, and after 10 min incubation. A control was prepared with buffer (Ferreres et al., 2021).

#### 2.6.4 Lipoxygenase (5-LOX) activity assay

The ability of the extracts and pure compounds to inhibit 5-LOX activity was assessed by measuring their capacity to prevent linoleic oxidation. In short, 200 μL of the extract solutions, diluted in 100 mM phosphate buffer (pH 9.0), was mixed with 20 μL of the enzyme solution and 20 μL of 4.18 mM linoleic acid prepared in ethanol, and the absorbance at 234 nm was measured. A control was prepared with buffer instead of sample (Trigueros, Oliveira, et al., 2024).

#### 2.6.5 Xanthine oxidase inhibition assay

Xanthine oxidase activity was determined using a previously established method by monitoring the conversion of xanthine to uric acid (Trigueros, Oliveira, et al., 2024). 200 μL of the extract solutions, diluted in 5 mM phosphate buffer (pH 7.4), was mixed with 10 μL of xanthine oxidase solution, incubated for 5 min at 37 °C, followed by the addition of 40 μL of 1.25 mM xanthine solution, and absorbance was measured at 295 nm. A control assay was prepared with buffer.

#### 2.6.6 Tyrosinase inhibition assay

To assess tyrosinase activity, 120 μL of the extract solutions, diluted in 50 mM phosphate buffer (pH 6.8), were combined with 40 μL of tyrosinase and 40 μL of 4.5 mM L-DOPA. The absorbance was measured at 475 nm to monitor L-DOPA oxidation. Buffer was used to prepare the control (Trigueros, Oliveira, et al., 2024).

#### 2.6.7 Acetylcholinesterase (AChE) inhibition assay

Acetylcholinesterase activity was assessed combining 75 μL of the extract solutions, prepared in 50 mM Tris-HCl buffer (pH 8.0), plus 0.1 % (w/*v*) albumin, with 25 μL of 15 mM ATCI and 125 μL of 3 mM DTNB. The formation of 5-thio-2-nitrobenzoate was monitored at 405 nm (Trigueros, Oliveira, et al., 2024).

### 2.7 Cellular assays

#### 2.7.1 Cell culture

Human gastric adenocarcinoma AGS, human colorectal adenocarcinoma Caco-2, human liver hepatocellular carcinoma HepG2, human neuroblastoma SH-SY5Y, and mouse microglia BV2 cells were obtained from ATCC (Spain). Cells were cultured in DMEM (1×) + GlutaMAX medium supplemented with 10 % (v/v) FBS and 1 % (v/v) penicillin/streptomycin in a humidified atmosphere with 5 % CO_2_ at 37 °C. They were passaged twice before use. Adherent cells, namely AGS, Caco-2, HepG2, and SH-SY5Y, were washed with HBSS upon reaching 70–80 % confluence and trypsinized for subculturing and seeded on 96-well plates at 15,000 cells/well for AGS, 20,000 cells/well for Caco-2, 10,000 cells/well for HepG2, and 40,000 cells/well for SH-SY5Y and BV2. The plates were incubated at 37 °C for 24 h.

#### 2.7.2 Cell viability assay

The impact of onion skin extracts on cell viability was evaluated using the MTT reduction assay (Ferreres et al., 2021). Subcultured cells were incubated with serial dilutions of the extract solution at 37 °C for 24 h. The extract solution was prepared by reconstituting the freeze-dried extracts in DMSO and diluting in culture medium to ensure a final DMSO concentrations ≤0.5 %, which was below the cytotoxic threshold. After incubation, the medium was aspirated and replaced with MTT. Absorbance was measured at 260 nm, and cell viability was calculated by comparing with a control, prepared with the same proportion of solvent used in the samples. At least three independent experiments were performed in triplicate.

#### 2.7.3 Inhibition of α-glucosidase in Caco-2 cells

The ability of onion skin extracts to inhibit the human α-glucosidase system, represented by the dimeric sucrase-isomaltase enzyme complex, was evaluated using a previously detailed method (Trigueros, Benito-Román, et al., 2024). First, the human enzyme system was obtained from cultured Caco-2 cells. Once the cells reached confluence, they were collected and disrupted using a glass/teflon Potter Elvehjem. The resulting homogenate was centrifugated, and the supernatant containing the dimeric sucrase-isomaltase enzyme system was collected and stored at −4 °C until use. The activity of the human α-glucosidase system was assessed by incubating 140 μL of the supernatant with 1 mM 4-nitrophenyl-α-D-glucopyranoside, both alone (control) and in the presence of serial concentrations of onion skin extracts. Enzyme activity was monitored by measuring the formation of 4-nitrophenol at 405 nm and 37 °C over 4 h. The protein content in the supernatant was measured to standardize enzyme activity for different experiments. Three independent assays were conducted using distinct cell passages. After determining the inhibitory effects on the sucrase-isomaltase system from human Caco-2 cell homogenates, the direct impact on adherent Caco-2 cells was also evaluated. Caco-2 cells were incubated with 4-nitrophenyl-α-D-glucopyranoside alone (control), and in the presence of the extract solution at the desired concentration, prepared as described for the cell viability assays. The extracts were tested at concentrations corresponding to their IC_50_ values from the previous assay. After 2 h incubation, medium was collected and the release of 4-nitrophenol was measured at 405 nm. Blanks were used to account for the intrinsic colour of cells and extracts, avoiding interference. Assays were conducted in triplicate using different cell passages.

#### 2.7.4 Inhibition of NO production in IFN-activated BV2 cells

After subculturing BV2 cells, serial dilutions of the extract solution were incubated at 37 °C, with two wells without sample as controls. After 2 h, interferon-γ (IFN) (0.864 mg/mL) was added to both extract-treated wells and one of the control wells, representing the IFN-activated BV2 cells. The other control, without IFN, was used to assess the basal NO production of the BV2 cells. Following a 24 h incubation, NO production was measured using the Griess reagent. 75 μL of each sample was mixed with 75 μL of Griess reagent, incubated in the dark for 10 min, and the absorbance was measured at 560 nm. The NO production in cells treated with onion skin extracts was estimated relatively to the control with IFN. Experiment was performed in triplicate using different cell passages.

### 2.8 Microbiological assays

#### 2.8.1 Fungal organisms

Three yeast strains (*Candida albicans* (ATCC 10231), *Candida krusei* (ATCC 6258), and *Cryptococcus neoformans* (CECT1078)) and filamentous fungi (one *Aspergillus fumigatus* (ATCC 204305) and three dermatophytes (*Trichophyton rubrum* (FF5), *Nannizzia gypsea* (formerly *Microsporum gypseum*-FF3) and *Epidermophyton floccosum* (FF9)) were used in this work. Sub-cultures were prepared in Sabouraud Dextrose Agar medium (SDA) from 1–2 days (yeasts and *Aspergillus*) to 5–7 days (dermatophytes) to ensure optimal growth conditions and purity.

#### 2.8.2 Assessment of antimicrobial activity

Fungal organisms were cultured in SDA and then suspended in RPMI-1640 broth. Serial dilutions of the onion skin extract solutions, prepared from a stock solution (8 mg/mL in DMSO), were prepared in RPMI-1640 broth, starting at a concentration of 4 mg/mL. Sample dilutions and cell suspensions were distributed into 96 well plates and incubated under aerobic, humid conditions at 26 °C for dermatophytes and 36 °C for the other organisms. Incubation times were 5 to 7 days for dermatophytes, 72 h for *C. neoformans*, and 48 h for the remaining organisms. After incubation, the Minimal Inhibitory Concentration (MIC) was determined as the lowest concentration showing 100 % inhibition of growth on the plates. In cases where MIC was determined, 20 μL from wells without visible growth were cultured on SDA plates, incubating under the same conditions previously described for each organism. The Minimal Fungicidal Concentration (MFC) was then determined as the lowest concentration where complete no growth was observed on the SDA plates. All the experiments were conducted in triplicate.

### 2.9 Statistical analysis

Statistical analysis was conducted using version GraphPad Prism 8.4.2. All results were presented as mean ± standard error of the mean (SEM). Student *t*-test was applied to assess significant differences with a significance level of *p* < 0.05 (*) and *p* < 0.01 (**).

## 3 Results and discussion

### 3.1 Chemical characterization of onion skin extracts

As a key part of this study, the composition of onion skin extracts was analyzed to establish a relationship between their chemical compounds and their biological effects. The primary components in both hydroalcoholic extracts were phenolics (103–155 mg/g), followed by flavonoids (81–115 mg/g), while sugars and proteins made up less than 10 % and 1 %, respectively (Table 1). Among the phenolic compounds, quercetin and quercetin-4’-*O*-glucoside were the most abundant, representing 61–67 % of the total phenolics. Other significant phenolics included protocatechuic acid (12–16 %), kaempferol (8–12 %), and *p*-coumaric acid (1–8 %), along with smaller amounts of myricetin, isorhamnetin, and other quercetin glucosides such as quercetin-3-4’-*O*-diglucoside and quercetin-3-*O*-glucoside. The corresponding chromatograms obtained by HPLC-DAD are depicted in Fig. S1. These findings align with those reported in literature. Celano et al. (2021) identified quercetin-4′-glucoside as the primary flavonoid in brown and red onion skin, followed by quercetin, although content was lower compared to the present study.

**Table 1:** Characterization of the ethanolic extracts derived from onion skin. Results are expressed in mg of each component per gram of extract in dry basis (db).

(mg/g, db)		*Horcal*	*Red*
Sugars		93 ± 6^a^	84 ± 4^a^
Proteins		5.6 ± 1.2^a^	9.0 ± 0.8^b^
Total Flavonoid Content (TFC)		115 ± 6^a^	81 ± 5^b^
Total Phenolic Content (TPC)		412 ± 5^a^	335 ± 7^b^
Phenolic compounds profile (HPLC-DAD):			
1	Protocatechuic acid	18.0 ± 0.2^a^	16.2 ± 0.1^b^
2	*p*-Coumaric acid	12.8 ± 0.9^a^	1.3 ± 0.1^b^
3	Quercetin-3,4´-*O*-diglucoside	2.7 ± 0.1^a^	6 ± 3^a^
4	Quercetin-3-*O*-glucoside	0.30 ± 0.01^a^	0.22 ± 0.05^a^
5	Quercetin-4´-*O*-glucoside	51.5 ± 0.3^a^	39 ± 1^b^
6	Myricetin	2.6 ± 0.3^a^	2.0 ± 0.2^b^
7	Quercetin	52 ± 2^a^	24 ± 1^b^
8	Kaempferol	12.6 ± 0.1^a^	12.2 ± 0.2^b^
9	Isorhamnetin	2.3 ± 0.1^a^	1.99 ± 0.01^b^

Data presented as mean ± SEM (*n* = 3). Significant differences were found between values with different letters in each colum (*p* < 0.05).

There were some differences in the chemical composition depending on the onion cultivar. The *Horcal* variety exhibited higher concentrations of most compounds, including sugars, TFC, TPC, and phenolic compounds, except for proteins. Specifically, sugars increased by 11 %, flavonoids by 42 %, and phenolics by 50 % in *Horcal*, while proteins decreased by 62 %. Regarding the phenolic profile, in general, *Horcal* showed higher concentrations than *Red*. For example, the quercetin content in *Horcal* was more than double, and the content of quercetin-4’-*O*-glucoside, protocatechuic acid, and kaempferol was 32 %, 11 %, and 3 % higher, respectively. Notably, the *p*-coumaric acid content was almost ten times higher in *Horcal* compared to *Red* onion variety. The flavonoid and phenolic composition of onion skin extracts can vary based on factors such as cultivar, origin, genetic variations, environmental conditions, sample preparation, and extraction methods, including solvent, temperature, and extraction procedure (Khalili et al., 2022). For example, the quercetin content in this study was 5–12 times higher than that reported by Messias et al. (2023) for white Valencian onions using ethanol Soxhlet extraction at 70 °C for 2 h. Additionally, onion skin is richer in flavonoids than the onion bulb or other edible parts. For example, red onion skin exhibited a TPC and TFC more than three and nearly 200 times higher, respectively, compared to red onion flesh (Albishi et al., 2013). After analyzing chemical composition of the extracts, quercetin, representing itself and its related glucosides, along with protocatechuic acid as predominant compounds accounting for more than 80 % of the total phenolic content, were selected for experimental assays. Assays were conducted under the same experimental conditions to elucidate their role in the observed biological effects.

### 3.2 Bioactive potential of onion skin extracts

#### 3.2.1 Antioxidant capacity

**Fig. 1:**
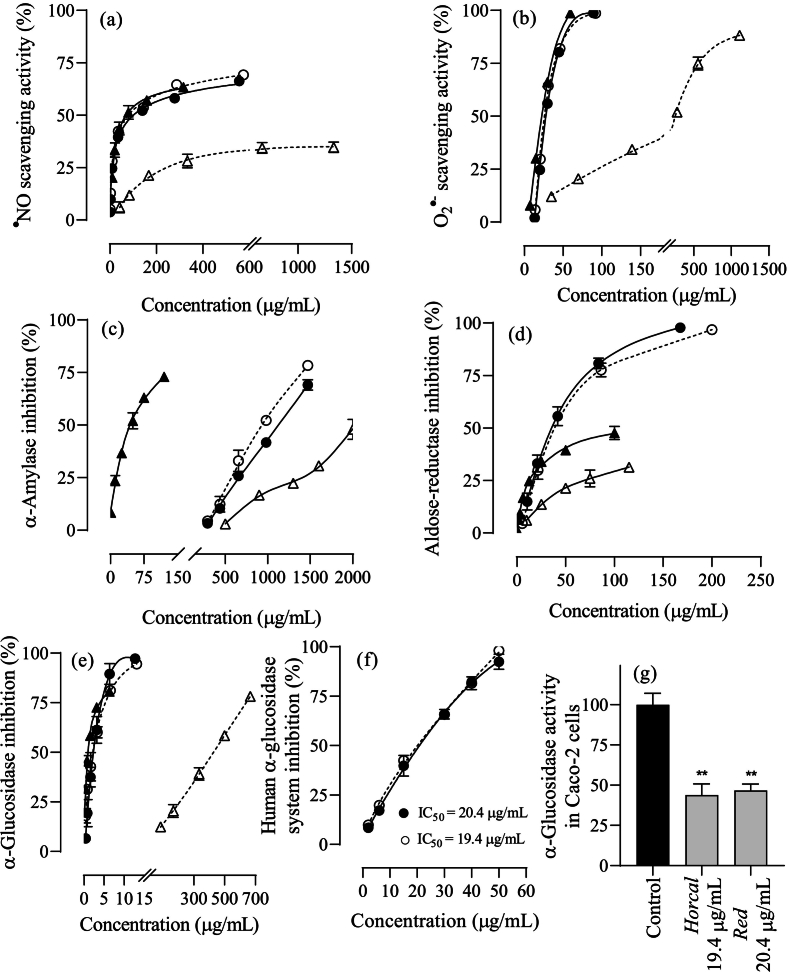
Effect of ethanolic aqueous extracts from onion skin of *Horcal* (○) and *Red* (●) cultivars, including quercetin (▲) and protocatechuic acid (△) as major compounds, on (a) nitric oxide and (b) superoxide anion radical scavenging activity, on (c) α-amylase, (d) aldose-reductase, and (e) α-glucosidase pure enzymes activity, and on α-glucosidase activity from (f) homogenates of human Caco2 cells and (g) adherent human Caco2 cells. Data are expressed as mean ± SEM (*n* = 3). *p* < 0.05 (*) and *p* < 0.01 (**). (For interpretation of the references to colour in this figure legend, the reader is referred to the web version of this article.)

Onion skin has been widely studied as a source of natural antioxidants, with quercetin being the most common flavonoid and exhibiting strong antioxidant activity. This study evaluated the antioxidant potential of onion skin extracts against oxidants responsible for causing damage to macromolecules in biological and food systems. For ^●^NO, all the extracts demonstrated scavenging below 75 % across the concentration range tested (Fig. 1a). Both the *Horcal* and *Red* varieties followed similar concentration-dependent trends, showing no significant difference in their IC_50_ values, which were 53 ± 3 μg/mL for *Horcal* and 56 ± 1 μg/mL for *Red* onion skin. Compared to quercetin, a major phenolic compound found in onion skin extracts, these values were similar, with an IC_50_ value determined for quercetin of 49 ± 2 μg/mL, while protocatechuic acid exhibited scavenging activities below 40 % at the maximum tested concentration (1.33 mg/mL), not being possible to estimate its IC_50_ value (IC_25_ = 237 ± 16 μg/mL). This suggests that quercetin, the primary phenolic compound along with its glucoside forms, contributed the most to the ^●^NO scavenging activity of the extracts, with other phenolic compounds contributing cumulatively. Regarding superoxide anion radical, all the extracts were significantly more active than for ^●^NO, with *Horcal* and *Red* onion skin extracts displaying similar trends (Fig. 1b), and no significant differences in their IC_50_ values: 26 ± 2 μg/mL for *Horcal* and 27.7 ± 0.5 μg/mL for *Red*. These values were close to quercetin (22.4 ± 1.1 μg/mL), with no significant differences between quercetin and *Horcal* extract. As with ^●^NO, protocatechuic acid was much less active than quercetin, exhibiting an IC_50_ value 11 times higher (252 ± 8 μg/mL), emphasizing quercetin key role in the antioxidant activity of the onion skin extracts. Hence, both *Horcal* and *Red* onion skin extracts proved effective scavenging potential toward oxidant species as O_2_^●-^ and ^●^NO, superior to previously reported literature, with quercetin mostly contributing.

According to the literature, both quercetin and protocatechuic acid are effective O_2_^●-^ scavengers in a concentration-dependant manner, with protocatechuic acid as a weaker scavenger, with a IC_50_ value (310 μg/mL) (Yuting et al., 1990) similar to that reported in this study. The superior scavenging power of quercetin could be attributed to its 3′-4′-diphenolic group, absent in protocatechuic acid, linked to high radical-scavenging efficiency (Yuting et al., 1990). Singh et al. (2009) observed that an ethanolic extract from red onion skin exhibited higher ^●^NO and O_2_^●-^ scavenging potential than butylated hydroxytoluene (BHT), with IC_50_ values of 200 μg/mL for both radicals, higher than those determined in this study. Most of the studies in literature employ non-biological radicals, such as DPPH and ABTS, which have no physiological relevance. For example, a study on red onion extracts found over 80 % DPPH neutralization at 100 μg/mL, but less than 45 % for ^●^NO at 1600 μg/mL (Khalili et al., 2022). Another study on protocatechuic acid revealed significantly different scavenging activities depending on the assay, with IC_50_ values of 1.88 μg/mL for DPPH, 0.89 μg/mL for ABTS, 74 μg/mL for hydroxyl radicals, and 310 μg/mL for superoxide anion radical (Li et al., 2011). This highlights the need to carefully evaluate the antioxidant activity, due to the high variability depending on target radical, and the opportunity of using these onion skin extracts to develop nutraceuticals and functional foods.

#### 3.2.2 Antidiabetic activity

The antidiabetic potential of onion skin extracts was assessed by evaluating their ability to inhibit key carbohydrate-digestion enzymes. Both extracts exhibited concentration-dependent inhibition across α-glucosidase, α-amylase, and aldose reductase, with the strongest effect on α-glucosidase, followed by aldose reductase and α-amylase (Fig. 1c-e). Nearly complete inhibition was achieved for α-glucosidase and aldose reductase, resulting in residual activity as low as 5 % at concentrations between 13 and 200 μg/mL, while α-amylase retained over 20 % activity at the highest concentration tested (1472 μg/mL). Moreover, no significant differences (*p* ≤ 0.05) were observed between onion varieties for α-glucosidase and aldose reductase inhibition assays. Both *Horcal* (IC_50_ = 44 ± 4 μg/mL) and *Red* (IC_50_ = 37 ± 4 μg/mL) onion skin extracts showed greater aldose reductase inhibition compared to quercetin (IC_50_ = 84 ± 6 μg/mL). For α-glucosidase, both extracts, with IC_50_ values of 2.2 ± 0.3 μg/mL for *Horcal* and 2.3 ± 0.5 μg/mL for *Red* variety, followed a similar inhibition pattern to quercetin (IC_50_ = 1.01 ± 0.06 μg/mL) (Fig. 1e) and were more effective than protocatechuic acid (IC_50_ = 425 ± 17 μg/mL) and acarbose (IC_50_ = 129.5 ± 1.0 μg/mL). Most α-glucosidase inhibitors do not interfere on mammalian α-glucosidase due to distinct enzyme recognition mechanisms (S. H. Kim et al., 2011). To enhance the relevance of the findings for developing functional foods for human consumption, the effects of onion skin extracts on α-glucosidase from human Caco2 cells were examined. Both extracts showed high inhibition against α-glucosidase from Caco2 homogenates (Fig. 1f, Fig. S2) and directly on adherent Caco2 cells (Fig. 1g), with IC_50_ values nine times higher than those determined for the pure enzyme from *S. cerevisiae*, reflecting the varied sensitivity of enzymes from different biological sources to inhibitors. While numerous studies highlight the antidiabetic properties of phenolic compounds in onions (Ren & Zhou, 2021), fewer focus specifically on onion skin. Nile et al. (2021) observed even lower inhibition in extracts from outer skins and onion bulb trimmings, with IC_50_ values 19–33 times higher. Moreover, the extracts obtained in this study also outperformed those previously reported for edible parts of onion (Kang et al., 2010). In a previous work on *Horcal* and *Red* onion skins, extracts obtained through subcritical water extraction exhibited weaker inhibition, with IC_50_ values 33–35 and 11–13 times higher for α-glucosidase and aldose reductase, respectively, compared to the current study. Additionally, these extracts showed reduced effectiveness against α-glucosidase in Caco2 cells, possibly due to lower TFC and TPC, reduced by 68–77 % and 21–31 %, respectively (Trigueros, Benito-Román, et al., 2024). Overall, onion skin extracts from *Horcal* and *Red* cultivars demonstrated significant hypoglycemic effects in both cellular and non-cellular models, surpassing those of other reported onion extracts and even exceeding the efficacy of the common oral antidiabetic acarbose. Additionally, due to their considerably low α-amylase inhibition, these extracts could be used for developing nutraceuticals avoiding gastrointestinal side effects associated with strong α-amylase inhibition and hypoglycemic medications (Trigueros, Benito-Román, et al., 2024).

#### 3.2.3 Anti-inflammatory activity

**Fig. 2:**
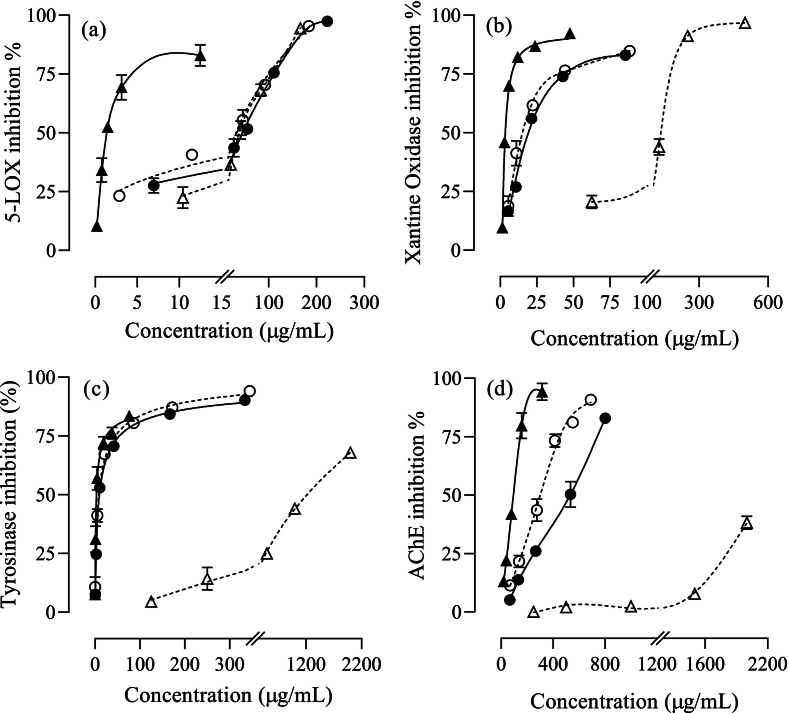
Inhibitory capacity of ethanolic aqueous extracts from onion skin of *Horcal* (○) and *Red* (●) cultivars, including quercetin (▲) and protocatechuic acid (△) as major compounds, on the activity of (a) 5-lypoxygenase, (b) xanthine oxidase, (c) tyrosinase, and (d) acetylcholinesterase enzymes. Data are expressed as mean ± SEM (*n* = 3). (For interpretation of the references to colour in this figure legend, the reader is referred to the web version of this article.)

Active compounds in onion skin have been previously studied for their anti-inflammatory properties. In this study, the anti-inflammatory potential of onion skin extracts was evaluated by measuring their capacity to inhibit 5-LOX. The results showed that both *Horcal* and *Red* onion cultivars exhibited a concentration-dependent inhibition, reaching nearly complete inhibition at the highest concentrations tested (95–97 % inhibition at 184–223 μg/mL) (Fig. 2a). However, the significantly lower (*p* < 0.05) IC_50_ value of the *Horcal* extract (30.2 ± 0.4 μg/mL) indicated higher activity compared to the *Red* extract (IC_50_ = 47 ± 8 μg/mL). Among the major phenolic compounds present in the extracts, pure quercetin showed significantly greater inhibition (IC_50_ = 1.41 ± 0.13 μg/mL) than protocatechuic acid (IC_50_ = 38 ± 3 μg/mL), with a concentration-dependent 5-LOX inhibition pattern like the exhibited by onion skin extracts. These findings are consistent with previous studies. For instance, Milea et al. (2020) reported a 49 % inhibition of lipoxygenase by flavonoids microencapsulated from yellow onion skin extracts, with an inhibition lower than observed in this study due to lower TPC and TFC. After confirming the anti-inflammatory potential of onion skin extracts through LOX inhibition, their capacity to inhibit NO production in IFN-activated microglial cells will be discussed (section 3.4).

#### 3.2.4 Other bioactivities

To gain deeper understanding of onion skin bioactive potential, inhibitory effects on various enzymes were studied. The ethanolic aqueous onion skin extracts demonstrated concentration-dependent inhibition, with stronger inhibition for xanthine oxidase and tyrosinase compared to acetylcholinesterase (Fig. 2b-d).

Xanthine oxidase plays a key role in human purine metabolism, catalyzing the oxidation of hypoxanthine to xanthine, which is converted to uric acid. Elevated serum uric acid levels can act as prooxidants, increasing ROS production and contributing to vascular damage. Inhibiting xanthine oxidase helps reduce serum uric acid levels, with benefits for cardiovascular health (Cicero et al., 2021). Both *Horcal* and *Red* onion skin extracts showed strong xanthine oxidase inhibitory activity, with over 75 % inhibition at the highest tested concentrations (86–88 μg/mL) (Fig. 2b), with *Horcal* demonstrating significantly higher activity with an IC_50_ value (15.1 ± 1.4 μg/mL) 21 % lower than *Red* (19.2 ± 0.9 μg/mL). Both extracts exhibited lower inhibitory capacity than quercetin (IC_50_ = 4.2 ± 0.6 μg/mL), overcame protocatechuic acid (IC_50_ = 140 ± 14 μg/mL), highlighting the relevance of onion skin flavonoids in the inhibition the 5-LOX enzyme. Nile et al. (2021) reported xanthine oxidase inhibitory potential of quercetin-4´-*O*-glucoside similar to quercetin, while quercetin-3-*O*-glucoside and quercetin-3,4´-*O*-diglucoside exhibited IC_50_ values 1.5 and 2 times higher. The higher inhibitory activity of the *Horcal* extract could be related to the higher content of quercetin and quercetin-4´-*O*-glucoside, 2.2 and 1.3 greater than *Red* extract (Table 1). Other authors reported similar IC_50_ values for onion derived extracts: 15.2–35.8 μg/mL for extracts obtained from edible onion outer layers and apical trimmings (S. H. Nile et al., 2017), 20.1–48.5 μg/mL for red onion skin and trimmings extracts (A. Nile et al., 2021), and 10.7 μg/mL for flavonols extracted from onion skin (J. S. Kim et al., 2023) observing a correlation between flavonols content and the decrease on xanthine oxidase activity, as observed in this study. Hence, these onion skin extracts offer potential as nutraceuticals for lowering serum uric acid levels *via* xanthine oxidase inhibition, providing a natural alternative to allopurinol, a common xanthine oxidase inhibitor associated with gastrointestinal, skin-related side effects, and hypersensitivity syndrome (Cicero et al., 2021).

Tyrosinase is a copper oxidase involved in melanin synthesis through a two-step process: first hydroxylating monophenols (monophenolase activity), then converting *o*-diphenols into *o*-quinones (diphenolase activity), leading to melanin formation. Overexpression of tyrosinase causes excess melanin production, contributing to pigmentation disorders (Chen & Kubo, 2002), being also linked to neurodegeneration in Parkinson's disease (Ma et al., 2024). Current approaches focus on replacing synthetic with natural tyrosinase inhibitors for medical, cosmetic, and food industry applications (Chen & Kubo, 2002). Flavonoids have gained attention as effective, non-toxic tyrosinase inhibitors, offering a safer alternative to synthetic options often associated with side effects like dermatitis, cytotoxicity, and carcinogenicity. Among flavonoids, quercetin has been described as the most potent tyrosinase inhibitor working by competitively binding copper chelation in the active center of the enzyme (Chen & Kubo, 2002). In this study, no significant differences were found between the IC_50_ values of the two cultivars, 14.9 ± 1.0 μg/mL for *Horcal* and 15.5 ± 0.5 μg/mL for *Red*, with remaining enzyme activity below 10 % at the highest tested concentrations (334–345 μg/mL). Moreover, onion skin extracts exhibited similar tyrosinase inhibition to quercetin (IC_50_ = 5.4 ± 1.5 μg/mL), while protocatechuic acid demonstrated lower activity (IC_50_ = 1218 ± 50 μg/mL) (Fig. 2c). This suggests that the inhibition capacity of the extracts was more strongly influenced by the presence of quercetin and related compounds. Furthermore, the tyrosinase inhibition capacity of the extracts was higher than other reported in the literature for onion extracts. For instance, in a study conducted with edible parts from two kazakh onions species extracted with absolute ethanol, 70 % ethanol and 50 % ethanol, the authors observed inhibition rates below 25 % at an extract concentration of 100 μg/mL (Kadyrbayeva et al., 2021), while in the present study inhibition above 75 % has been observed at the same concentration. Another study on methanol extracts from various *Allium* species reported IC_50_ values ranging from 64 μg/mL to 11.87 mg/mL (Özel et al., 2024), with the lowest value being four times higher than those determined in this study. Additionally, Nile et al. (2021) obtained 80 % methanol extracts from red onion samples, including outer skins and trimmings, with IC_50_ values ranging from 38.9 to 65.9 μg/mL, higher than in this study.

In Alzheimer's disease, the neurotransmitter acetylcholine, which plays a crucial role in cognitive functions regulation, is significantly reduced. Acetylcholinesterase (AChE) is the primary enzyme that controls acetylcholine levels, making AChE inhibitors valuable for enhancing brain cholinergic connectivity by increasing both the level and duration of the neurotransmitter action (Park et al., 2015). The potential of onion skin extracts to inhibit AChE activity varied between the *Horcal* and *Red* varieties, with the *Horcal* showing greater potency (IC_50_ = 301 ± 20 μg/mL) (Fig. 2d), as indicated by a significantly 43 % lower (*p* < 0.05) IC_50_ value compared to *Red* (IC_50_ = 532 ± 51 μg/mL). This greater potential aligns with its higher TPC and TFC (Table 1). As previously mentioned, onion skin extracts exhibited a weaker AChE inhibition compared to xanthine oxidase and tyrosinase, a trend that was also observed for quercetin and protocatechuic acid. Specifically, for protocatechuic acid, displaying the weakest inhibitory effect, its IC_50_ could not be determined (IC_25_ = 1809 ± 31 μg/mL). Additionally, the extracts demonstrated lower activity than quercetin (IC_50_ = 94 ± 4 μg/mL), indicating that their potential was influenced not only by quercetin but also by other compounds besides protocatechuic acid, such as quercetin-4´-*O*-glucoside and quercetin-3,4´-*O*-diglucoside. There are limited studies on the ability of onion extracts to reduce AChE activity. Park et al. (2015) reported IC_50_ values for onion flesh extracts that were similar to those found in this study, while the values for onion peel extracts were lower, though no information was provided regarding the concentration of main phenolics. Consistently with this study, Özel et al. (2024) also found that methanol extracts from various *Allium* species had lower AChE inhibitory potential compared to α-glucosidase and tyrosinase, with reported IC_50_ values ranging from 0.335 to 4.651 mg/mL, which are significantly higher than those found in this study. The relationship between Alzheimer's disease, inflammation, and oxidative stress has been well-documented. An imbalance between antioxidant defenses and oxidative stress can lead to inflammation, which may result in neuroinflammation and neurodegeneration. These findings suggest that onion skin extracts, containing moderate AChE inhibitors and combined with strong antioxidant and anti-inflammatory properties, could serve as promising nutraceuticals and bioactive-compounds source.

### 3.3 Cytotoxicity of onion skin extracts

After confirming the bioactivity potential of onion skin extracts, their safety on human cells was evaluated using AGS, Caco-2, and HepG2 cell lines, commonly used to assess bioactive compounds effects in the gastrointestinal tract (Trigueros, Oliveira, et al., 2024). Despite onion peels exhibiting anti-cancer properties in the literature (Kumar et al., 2022), neither *Horcal* nor *Red* onion skin extracts caused significant changes (*p* ≤ 0.05) in cell viability at the tested concentrations (Fig. S3). The concentrations, ranging from 2.09 to 552 μg/mL, were based on the bioactivity of the extracts, except for α-amylase for which enzyme activity is desired to be maintained to avoid side effects. Thus, onion skin extracts were shown to be safe, as no changes in mitochondrial-dependent MTT reduction were observed in any of the cell lines. Few studies have examined onion bulb effects on HepG2 and Caco-2 cells. Pan et al. (2018) reported no significant effects on HepG2, HT-29, and PC-3 cell lines. Similarly, in a previous work, *Horcal* and *Red* onion skin extracts showed no effects on AGS, Caco-2, and HepG2 cells, though lower concentrations than in this study were evaluated (Trigueros, Benito-Román, et al., 2024). These results suggest that onion extracts either do not affect cell viability or require higher concentrations to induce cytotoxicity, confirming their safe profile within the tested concentration range where they exert their main bioactive potential. Additionally, onion skin extracts were tested for neurotoxic effects on SH-SY5Y neuronal cells. Neither *Horcal* nor *Red* onion skin extracts caused significant changes in mitochondrial activity in these cells at tested concentrations (Fig. S3g-h). Similar results were found for extracts from spent coffee grounds, containing *p*-coumaric acid and quercetin (Angeloni et al., 2021).

### 3.4 Anti-neuroinflammatory activity of onion skin extracts

**Fig. 3:**
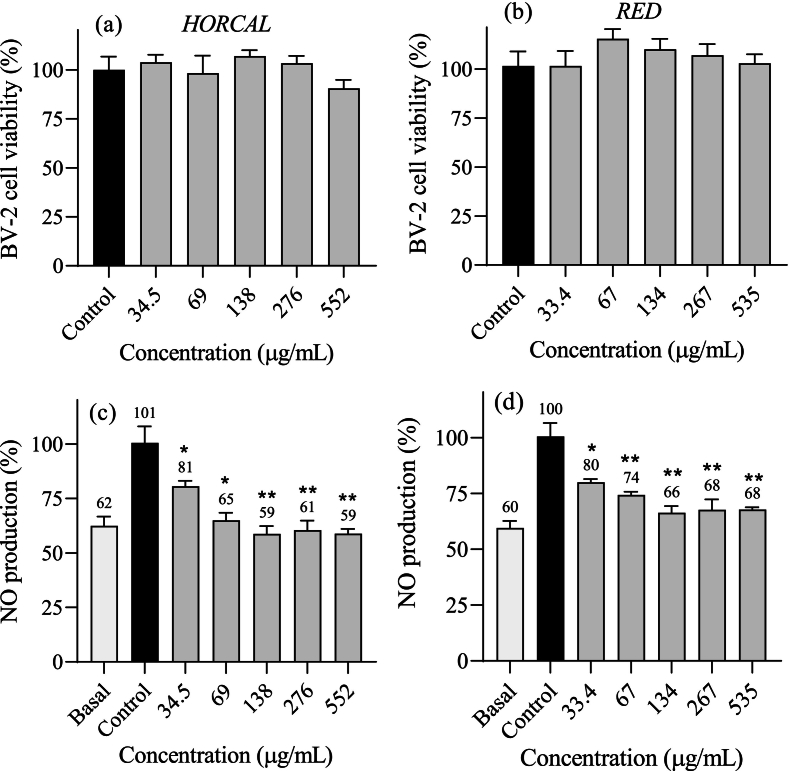
Impact of ethanolic aqueous onion skin extracts from (a,c) *Horcal* and (b,d) *Red* cultivars on the viability of BV-2 cell lines and on NO levels in IFN-activated BV-2 cells treated with serial dilutions. ‘Basal’ refers to NO production in BV2 cells without IFN, while ‘Control’ refers to NO production in IFN-activated BV2 cells. Data are expressed as mean ± SEM (*n* = 3). *p* < 0.05 (*) and *p* < 0.01 (**). (For interpretation of the references to colour in this figure legend, the reader is referred to the web version of this article.)

The underlying mechanism of neuroinflammation involves microglia activation in response to inflammatory agents, leading to the production of ROS and NO. While NO acts as neuromodulator at normal levels, elevated NO production contributes to oxidative stress and neuronal damage, ultimately resulting in neurodegeneration. As a result, natural products that can modulate NO production are of interest. In this study, the impact of onion skin extracts on NO production in IFN-activated BV-2 cells was examined. Before assessing their effects on neuroinflammation, the cytotoxicity of onion skin extracts was evaluated in BV-2 microglia cells. None of the tested concentrations showed cytotoxic effects (Fig. 3a-b), consistent with previous studies (Jakaria et al., 2019). These findings further support the safe profile of onion skin extracts. After IFN stimulation, NO production in BV-2 cells increased up to 100–101 % compared to the non-activated basal state (60–62 %). Treatment with onion skin extracts significantly reduced NO production in activated microglial cells (Fig. 3c-d). Both *Horcal* and *Red* onion extracts led to a significant 20 % reduction (*p <* 0.05) in NO production at the lowest tested concentration (33.4–34.5 μg/mL). *Horcal* extract achieved greater NO reduction, restoring NO levels to the basal state at concentrations above 69 μg/mL. In contrast, NO production in cells treated with *Red* onion skin extract remained 6–8 % above the basal state at concentrations higher than 134 μg/mL. This difference may be attributed to the higher quercetin and quercetin-4´-*O*-glucoside content in the *Horcal* extract, as quercetin has been shown to effectively reduce NO production in LPS-induced BV-2 cells at concentrations above 5 μM (Sun et al., 2015). To the best of our knowledge, this is the first study to evaluate the anti-neuroinflammatory activity of onion skin extracts, contributing to a deeper understanding of the role of their flavonoids in modulating neuroinflammation, and highlighting the potential of onion skin extracts to prevent neuroinflammation in activated microglial cells, showing promise as a bioactive-compound source for functional foods development.

### 3.5 Antimicrobial activity of onion skin extracts

Tyrosinase, in addition to its role in melanin biosynthesis, is involved in spore formation within fungal pathways and plays a part in defense mechanisms against external factors. Given the tyrosinase inhibitory properties of onion skin extracts, their antimicrobial effects were evaluated, as shown in Table 2. The most resistant strains to the onion skin extracts were the yeasts *C. albicans*, *C. krusei*, and *C. neoformans*, along with the filamentous fungus *A. fumigatus*. However, their anti-dermatophytic effects were pronounced, especially against *T. rubrum* and *E. floccosum*. The MIC and MFC values for the human pathogenic yeasts *C. albicans* and *C. krusei* exceeded 4 mg/mL, consistent with the effects of quercetin and protocatechuic acid. For *C. neoformans* a MIC of 4 mg/mL was found for *Horcal*, linked to the higher quercetin and protocatechuic acid content (Table 1). These findings are consistent with previous reports. Sachikonye et al. (2016) revealed no significant growth inhibition by quercetin for these three yeasts but did observe increased effectiveness when using combined with other drugs against *C. albicans* and *C. neoformans* (Baqer et al., 2024). For *C. albicans*, the results align with previous studies, which reported MIC values between 1.56 and 23.20 mg/mL and MFC values between 12.50 and 50 mg/mL for bulb onion and onion skin extracts (Mounir et al., 2023). Variations were also observed in the effects of onion bulb extracts on *C. krusei*, with MICs ranging from 1 to 8 mg/mL and MFCs from 2 to 16 mg/mL, depending on the variety (Kadyrbayeva et al., 2021). Similar to yeasts, the effect of onion skin extracts on *A. fumigatus* growth was minimal, with MIC and MFC values above 4 mg/mL. Although no previous studies have reported the effects of onion skin extracts on *A. fumigatus*, lower MIC and MFC values have been found for onion bulb extracts, due to the presence of organosulfur compounds such as allicin and ajoene, which are absent in onion skin extracts (Yin & Tsao, 1999). For dermatophyte fungi, *T. rubrum* was the most sensitive to the onion skin extracts, with both MIC and MFC values of 1000 μg/mL for both varieties. This was followed by *E. floccosum*, which showed lower resistance to the *Horcal* extract, and *N. gypsea*, with a MIC of 4000 μg/mL (MIC_90_ = 2000 μg/mL for *Horcal*) but higher MFC. To the best of our knowledge, no studies have specifically reported the effects of onion skin on these dermatophytes, having demonstrated strong anti-dermatophyte activity, highlighting their potential as nutraceuticals for dermatophyte infections.

**Table 2:** Antifungal activity of ethanolic aqueous extracts from onion skin of *Horcal* and *Red* cultivars, including quercetin and protocatechuic acid as major compounds (see Table 1), tested against three yeast strains species, and four filamentous fungi (*A. fumigatus*, and three dermatophytes), and assessed by MIC (Minimal Inhibitory Concentration) and MFC (Minimal Fungicidal Concentration) values.

		MIC (μg/mL)^⁎1^				MFC (μg/mL)			
		*Horcal*	*Red*	Quercetin	Protocatechuic acid	*Horcal*	*Red*	Quercetin	Protocatechuic acid
Yeast strains	*Candida albicans*	>4000	>4000	>4000	>4000	>4000	>4000	>4000	>4000
	*Candida krusei*	>4000	>4000	≥4000	>4000	>4000	>4000	>4000	>4000
	*Cryptotoccus neoformans*	4000	>4000	1000 (750–90 %)	1000 ≥ 95 %	>4000	>4000	2000	2000
Filamentous fungi	*Aspergillus fumigatus*	>4000	>4000	>4000	>4000	>4000	>4000	>4000	>4000
	*Trichophyton rubrum*	1000	1000	1000	2000 (1000–90 %)	1000	1000	1000	2000
	*Nannizzia gypsea*	4000 (2000–90 %)	4000	>4000	>4000	>4000	>4000	>4000	>4000
	*Epidermophyton floccosum*	1000	2000	500	500	1000	2000	1000	2000

^⁎1^MIC is referred to the concentration causing 100 % growth inhibition, except in clearly specified cases.

## 4 Conclusions

Onion skin has been shown to be a rich source of bioactive compounds, particularly phenolic compounds, primarily composed of quercetin, its glucosides, and phenolic acids such as protocatechuic acid, with slight differences between *Horcal* and *Red* varieties. Flavonoid-rich extracts from onion skin have demonstrated various biological activities, including strong antioxidant effects against biological radicals, as well as significant antidiabetic and anti-inflammatory properties, proved in both cell and non-cell systems, with no significant differences between varieties. Additionally, these extracts effectively inhibited enzymes associated with elevated levels of uric acid (xanthine oxidase), melanin (tyrosinase), and acetylcholine (acetylcholinesterase), as well as demonstrating strong anti-dermatophytic effects. Finally, the non-cytotoxic nature of the extracts highlights onion skin as an excellent raw material for obtaining natural compounds with excellent bioactive potential that can be used in food supplements, functional foods, and nutraceuticals.

## CRediT authorship contribution statement

**Esther Trigueros:** Writing – review & editing, Writing – original draft, Project administration, Investigation, Formal analysis, Data curation, Conceptualization. **Óscar Benito-Román:** Writing – review & editing, Investigation, Funding acquisition. **Andreia P. Oliveira:** Writing – review & editing, Validation. **Romeu A. Videira:** Writing – review & editing. **Eugénia Pinto:** Writing – review & editing, Funding acquisition, Data curation. **Paula B. Andrade:** Validation, Funding acquisition, Formal analysis. **M. Teresa Sanz:** Writing – review & editing, Funding acquisition, Conceptualization. **Sagrario Beltrán:** Writing – review & editing, Funding acquisition.

## Funding

This work received financial support from the PT national funds (FCT/MCTES, (Fundação para a Ciência e Tecnologia and 10.13039/501100006111Ministério da Ciência, Tecnologia e Ensino Superior) through the project UIDP/50006 – Laboratório Associado para a Química Verde – Tecnologias e Processos Limpos.

## Declaration of competing interest

The authors declare that they have no known competing financial interests or personal relationships that could have appeared to influence the work reported in this paper.

## Data Availability

No data was used for the research described in the article.

## Appendix A Supplementary data

Supplementary material. Image 1
