# Supplementary material for: Non-edible onion skin waste as a source of bioactive agents for functional foods development: Chemical composition and multifunctional bioactivity
Source: Food Chem X. 2025 Jul 17;29:102794. doi: 10.1016/j.fochx.2025.102794 (PMC12320693; doi:10.1016/j.fochx.2025.102794)
Supplement: Supplementary file 1 — Supplementary material. [file mmc1.docx]

**SUPPLEMENTARY MATERIAL. Non-edible Onion Skin Waste as a Source of Bioactive Agents for Functional Foods Development: Chemical Composition and Multifunctional Bioactivity**

Esther Trigueros^a,b,*^, Óscar Benito-Román^b^, Andreia P. Oliveira^a,c,d^, Romeu A. Videira^a^, Eugénia Pinto^e,f^, Paula B. Andrade^a^, M. Teresa Sanz^b^, Sagrario Beltrán^b^

^a^REQUIMTE/LAQV, *Laboratório de Farmacognosia, Departamento de Química, Faculdade de Farmácia, Universidade do Porto, R. Jorge Viterbo Ferreira, nº 228, 4050-313 Porto, Portugal*.

^b^*Department of Biotechnology and Food Science, Chemical Engineering Division, University of Burgos, Plza. Misael Bañuelos s/n 09001 Burgos, Spain.*

^c^*Associate Laboratory i4HB – Institute for Health and Bioeconomy,* *University Institute of Health Sciences – IUCS-CESPU, 4585-116 Gandra, Portugal*

^d^*UCIBIO – Research Unit on Applied Molecular Biosciences, Translational Toxicology Research Laboratory, University Institute of Health Sciences (1H-TOXRUN, IUCS-CESPU), 4585-116 Gandra, Portugal*

*^e^Laboratory of Microbiology, Biological Sciences Department, Faculty of Pharmacy, University of Porto, 4050-313 Porto, Portugal*

*^f^Interdisciplinary Centre of Marine and Environmental Research (CIIMAR), University of Porto, 4450-208 Matosinhos, Portugal*

Esther Trigueros: [etrigueros@ubu.es](mailto:etrigueros@ubu.es); Óscar Benito-Román: [obenito@ubu.es](mailto:obenito@ubu.es); Andreia P. Oliveira: [asoliveira@ff.up.pt](mailto:asoliveira@ff.up.pt); Romeu A. Videira: [rvideira@ff.up.pt](mailto:rvideira@ff.up.pt); Eugénia Pinto: [epinto@ff.up.pt](mailto:epinto@ff.up.pt); Paula B. Andrade: [pandrade@ff.up.pt](mailto:pandrade@ff.up.pt); M. Teresa Sanz: [tersanz@ubu.es](mailto:tersanz@ubu.es); Sagrario Beltrán: [beltran@ubu.es](mailto:beltran@ubu.es)


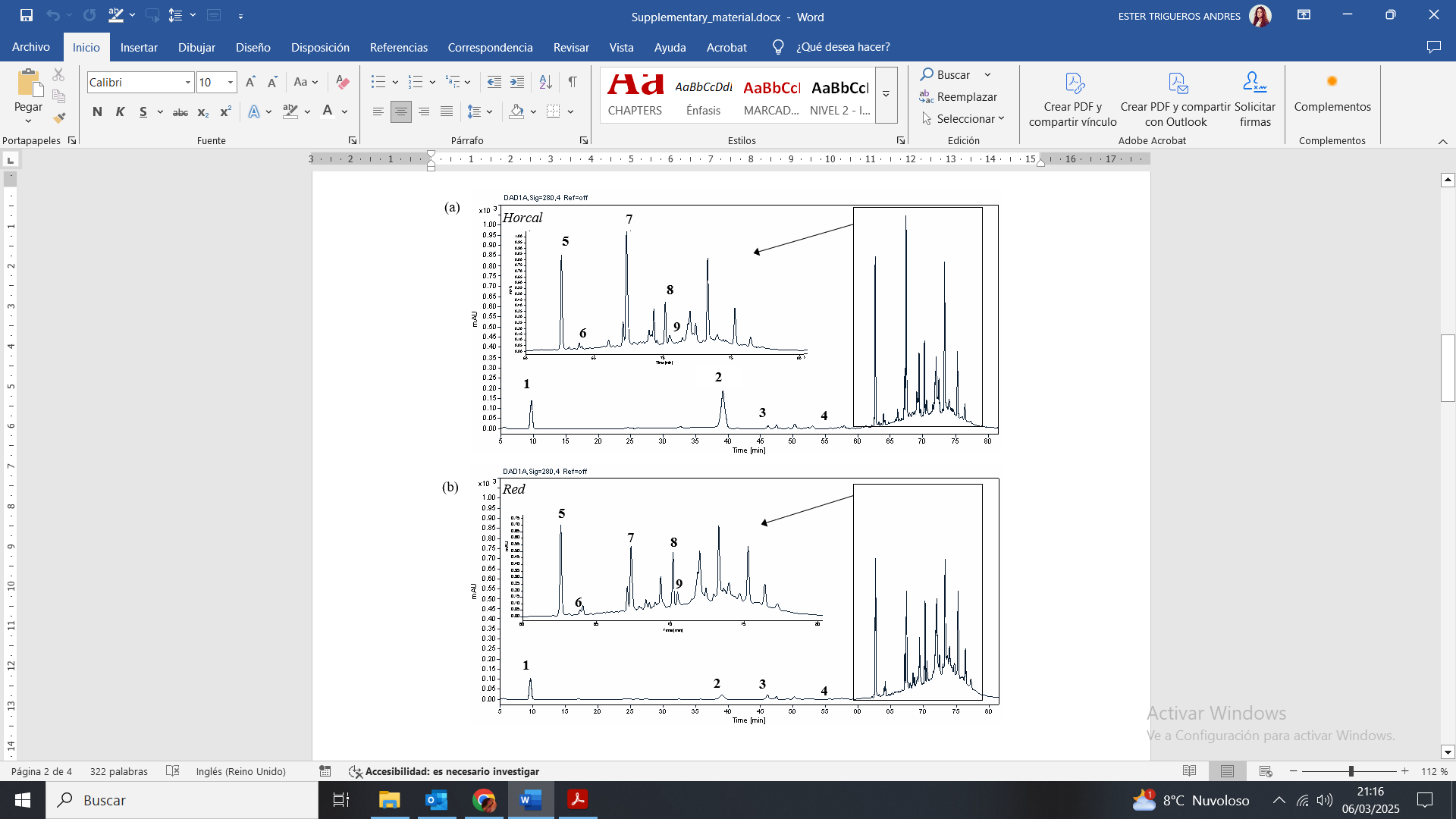


Figure S1. Chromatograms obtained via HPLC-DAD from ethanolic aqueous extracts of onion skin waste from (a) *Horcal* and (b) *Red* onion cultivars. The identification of the numbered peaks is detailed in Table 1.

Figure S2. Kinetics of 4-nitrophenyl-α-D-glucopyranoside (substrate) conversion in the absence and the presence of various concentrations of ethanolic aqueous extracts obtained of onion skin waste from (a)*Horcal* and (b) *Red* onion cultivars. These kinetics were used to estimate the inhibition of α-glucosidase derived from Caco-2 cells homogenates.

**Figure S3.** Impact of ethanolic aqueous extracts from onion skin of *Horcal* (first column) and *Red* (second column) cultivars on the viability of AGS, Caco-2, HepG2, and SH-SY5Y cell lines. Data are expressed as mean ± SEM (*n* = 3). *p* < 0.05 (*) and *p* < 0.01 (**).
